# Alisporivir Improves Mitochondrial Function in Skeletal Muscle of *mdx* Mice but Suppresses Mitochondrial Dynamics and Biogenesis

**DOI:** 10.3390/ijms22189780

**Published:** 2021-09-10

**Authors:** Mikhail V. Dubinin, Vlada S. Starinets, Eugeny Yu. Talanov, Irina B. Mikheeva, Natalia V. Belosludtseva, Konstantin N. Belosludtsev

**Affiliations:** 1 Department of Biochemistry, Cell Biology and Microbiology, Mari State University, 424001 Yoshkar-Ola, Russia; vlastar@list.ru (V.S.S.); bekonik@gmail.com (K.N.B.); 2 Laboratory of Mitochondrial Transport, Institute of Theoretical and Experimental Biophysics, Russian Academy of Sciences, 142290 Pushchino, Russia; evg-talanov@yandex.ru (E.Y.T.); mikheirina@yandex.ru (I.B.M.); nata.imagination@gmail.com (N.V.B.)

**Keywords:** Duchenne muscular dystrophy, skeletal muscle, alisporivir, Debio-025, mitochondria, cyclophilin D, mitochondrial permeability transition

## Abstract

Mitigation of calcium-dependent destruction of skeletal muscle mitochondria is considered as a promising adjunctive therapy in Duchenne muscular dystrophy (DMD). In this work, we study the effect of intraperitoneal administration of a non-immunosuppressive inhibitor of calcium-dependent mitochondrial permeability transition (MPT) pore alisporivir on the state of skeletal muscles and the functioning of mitochondria in dystrophin-deficient *mdx* mice. We show that treatment with alisporivir reduces inflammation and improves muscle function in *mdx* mice. These effects of alisporivir were associated with an improvement in the ultrastructure of mitochondria, normalization of respiration and oxidative phosphorylation, and a decrease in lipid peroxidation, due to suppression of MPT pore opening and an improvement in calcium homeostasis. The action of alisporivir was associated with suppression of the activity of cyclophilin D and a decrease in its expression in skeletal muscles. This was observed in both *mdx* mice and wild-type animals. At the same time, alisporivir suppressed mitochondrial biogenesis, assessed by the expression of *Ppargc1a*, and altered the dynamics of organelles, inhibiting both DRP1-mediated fission and MFN2-associated fusion of mitochondria. The article discusses the effects of alisporivir administration and cyclophilin D inhibition on mitochondrial reprogramming and networking in DMD and the consequences of this therapy on skeletal muscle health.

## 1. Introduction

Muscle hereditary diseases are rare, but rather severe, pathologies. One of these pathologies is Duchenne muscular dystrophy (DMD), caused by mutations in the gene encoding the dystrophin protein in muscles. This protein provides a link between the cytoskeleton and the sarcolemma, as well as other muscle proteins, by forming the dystrophin-associated glycoprotein complex, playing a key role in muscle contraction. Disorganization of this structure leads to dysfunction of muscle tissue, the development of progressive muscle wasting and weakness, and in the later stages is also associated with heart failure [1].

It is known that the structural instability of myocytes in DMD is accompanied by an increase in the permeability of the sarcolemma for calcium ions and a significant increase in the concentration of this ion in muscle fibers, which is accompanied by the activation of proteases and lipases and subsequent degradation of muscle tissue [1,2]. Normally, the calcium level in the cell is regulated due to the functional interaction of the sarcoplasmic reticulum and mitochondria, the main calcium depots of skeletal muscles [1,3]. Under DMD conditions, these organelles undergo significant reprogramming, leading to reduced ATP levels in skeletal muscle in both DMD patients and dystrophin-deficient animal models, as well as a significant decrease in the ability to accumulate excess calcium ions [4,5,6,7,8,9]. This, in turn, results in dysfunction of the contractile apparatus, leading to reduced muscular strength, dysregulation of intracellular Ca^2+^ buffering, loss of homeostasis, and rapidly progressive Ca^2+^-induced degeneration of skeletal muscle.

A decrease in the calcium-accumulating ability of skeletal muscle mitochondria in DMD is known to be due to the suppression of their functional activity, as well as to the fact that organelles become less resistant to the opening of the calcium-dependent MPT pore, a non-selective protein channel in the inner and outer mitochondrial membranes, that is permeable to molecules less than 1.5 kDa in size [8,9,10,11]. The induction of MPT is accompanied by a collapse of the membrane potential, disruption of ionic homeostasis, up to the destruction of mitochondria, and the release of protein factors that cause necrosis and apoptosis of muscle fibers, contributing to the development of generalized inflammation. The opening of the MPT pore occurs in the case of excessive accumulation of Ca^2+^ in the mitochondrial matrix, leading to the activation of cyclophilin D protein—peptidyl-prolyl cis-trans isomerase, playing an important role in initiating the assembly of the MPT pore channel. Adenine nucleotide translocator isoforms and ATP synthase are considered proteins involved in the formation of the pore channel [10]. We have previously shown that in the case of DMD, the channel protein may be represented by ANT2 protein, whose content significantly increases in the skeletal muscles of dystrophin-deficient *mdx* mice [8,9]. Nevertheless, one should recognize that the only protein that is currently identified as part of the MPT pore is the regulatory protein cyclophilin D, and studies on the role of the pore in cellular pathologies, including hereditary muscular dystrophies, focus precisely on its pharmacological and genetic modifications [12,13]. Indeed, the knockout of the cyclophilin D gene is known to successfully prevent mitochondrial dysfunction and the development of destructive processes in skeletal muscles in several models of muscular dystrophies including DMD [14]. Pharmacological inhibition of cyclophilin D activity by specific suppressor cyclosporin A (CsA), and its analog alisporivir (also known as Debio025) or isoxazoles (as TR001) also has a positive effect [6,15,16,17]. The beneficial effects of these agents are noted at the mitochondrial level and are also manifested in the improvement of pathology in preclinical animal models, a decrease in the level of fibrosis in skeletal muscles, mitigation of necrosis, and the inflammatory process intensity.

It is known that CsA, as the most famous inhibitor of cyclophilin D, is able to suppress the immune system, as well as inhibit calcineurin signaling secondarily reducing myotube differentiation and muscle regeneration [18]. Indeed, some negative effects of CsA therapy on the progression of DMD have been noted in *sapje* zebrafish and other animal models [6,18] and in DMD patients [6]. Therefore, the preference is now given to the non-immunosuppressive analog of CsA—alisporivir [19], which has shown promising effects in both animal models and muscle biopsies from DMD patients.

In this work, we continue to study the effect of alisporivir administration (intraperitoneally, 5 mg/kg per day) on the state of the skeletal muscles of dystrophin-deficient *mdx* mice and evaluate the morphological and functional changes in the mitochondria of this tissue, as well as the degree of reprogramming of the systems responsible for OXPHOS, MPT pore opening, dynamics and biogenesis of organelles. The results obtained indicate that alisporivir normalizes the state of the skeletal muscles of *mdx* mice, as well as the functional activity of mitochondria, but at the same time suppresses organelle dynamics and biogenesis.

## 2. Results

### 2.1. Alisporivir Reduces the Intensity of the Inflammatory Process and Improves the Muscle Function of Dystrophin-Deficient Mice

**Figure 1:**
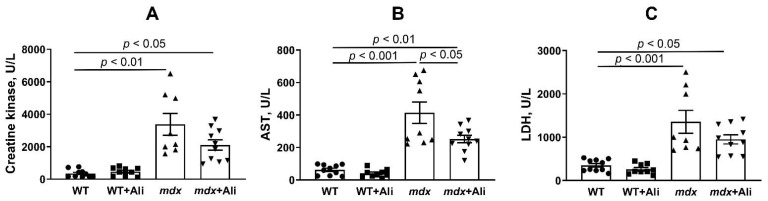
The effect of alisporivir treatment on the activity of creatine kinase (**A**), AST (**B**), and LDH (**C**) in the serum of experimental groups of mice. The data are presented as means ± SEM (*n* = 8–10).

It is known that dystrophin-deficient animals, as well as DMD patients, show a generalized inflammatory process in the skeletal muscles due to the destruction of cell membranes and the development of a necrotic process. One of the primary diagnostic criteria for assessing the intensity of inflammation is an increase in the level of marker serum enzymes, such as creatine kinase, aspartate aminotransferase (AST), and lactate dehydrogenase (LDH), which are normally localized inside cells and are massively released upon cell membrane disruption [15,16]. Indeed, one can see that the activity of these enzymes significantly increases in *mdx* mice, compared to healthy wild-type mice (Figure 1). In this case, alisporivir treatment is accompanied by a tendency toward a decrease in the level of creatine kinase and LDH, as well as a significant reduction in the level of AST in the serum of *mdx* mice, indicating alleviation of destructive processes in the skeletal muscles of DMD animals.

**Figure 2:**
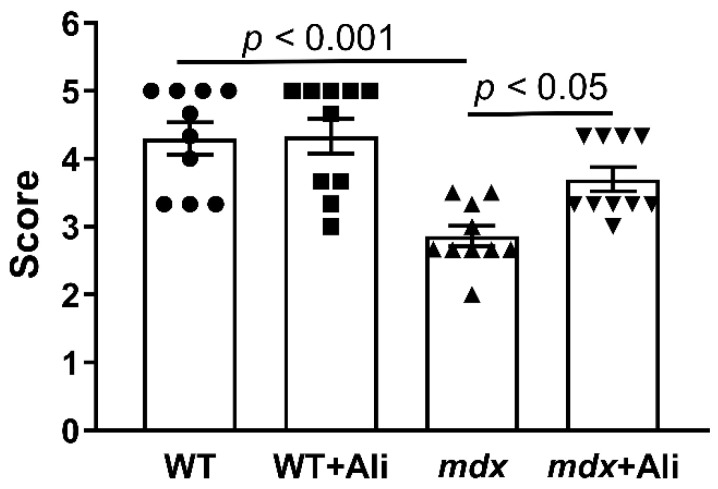
The results of a wire hang test, reflecting muscle function and endurance of the studied groups of mice. The data are presented as means ± SEM (*n* = 10).

In addition, we evaluated the effect of alisporivir on muscle function in experimental groups of animals. Figure 2 shows the results of a simple physiological test, indicating a strong decrease in the endurance of dystrophin-deficient animals, compared to healthy WT mice. Alisporivir-treated *mdx* mice show a significant improvement in muscle function, compared to control *mdx* mice.

**Figure 3:**
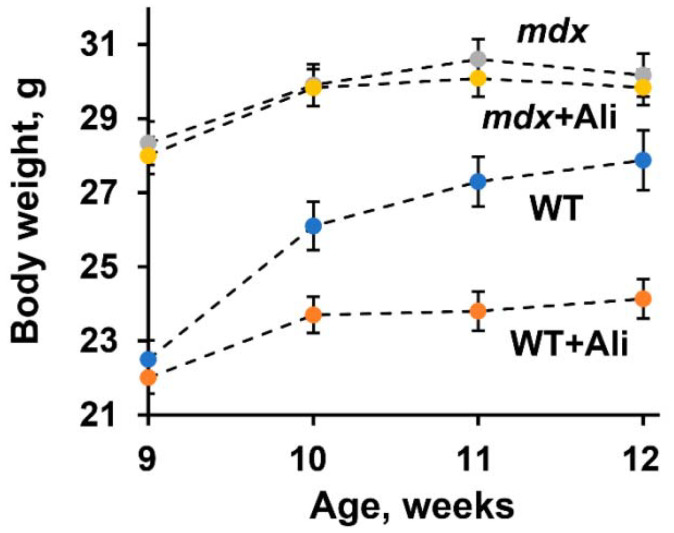
The effect of alisporivir treatment on body weight gain in experimental groups of mice. The data are presented as means ± SEM (*n* = 10).

Along with this result, we noted the effect of alisporivir on weight gain in animals during the experiment. One can see that initially, *mdx* animals show significantly higher body weight compared to wild-type animals of the same age, which is known to be due to the early development of pseudohypertrophy of skeletal muscles in dystrophin-deficient animals [16]. In this case, alisporivir administration tends to decrease the body weight of *mdx* mice, but this effect is most pronounced in the case of WT animals, whose body weight gain has significantly decreased (Figure 3).

These results generally confirm the data of other groups and indicate the beneficial effect of alisporivir on the structural integrity and functional state of muscles in Duchenne dystrophy [6,15,16]. However, in the case of wild-type mice, alisporivir significantly affects the muscle gain and growth of the animals.

### 2.2. The Effect of Alisporivir on the Ultrastructure of Skeletal Muscle Mitochondria

**Figure 4:**
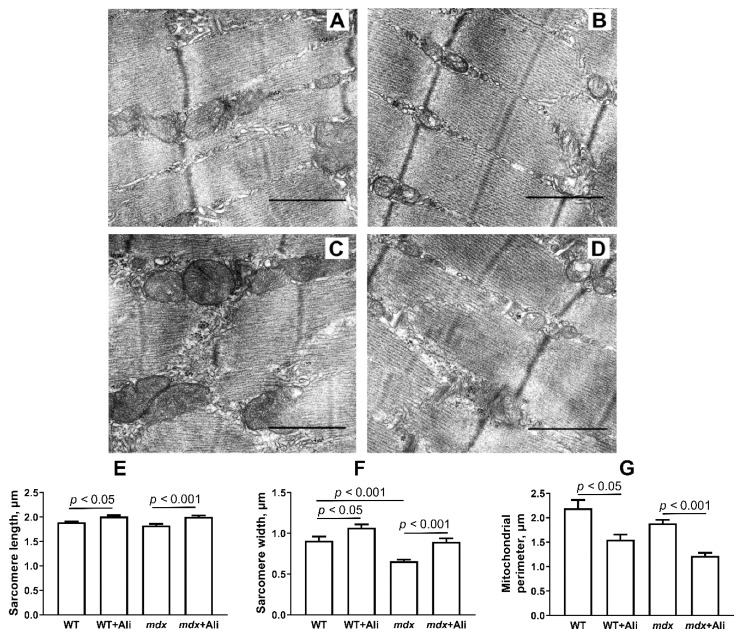
Representative electron micrographs of skeletal muscle tissue (quadriceps) from WT (**A**), WT+Ali (**B**), *mdx* (**C**), and *mdx*+Ali (**D**) groups of mice. The bar is equal to 1 μm. Graphical representation of micrograph profiles: sarcomere length in μm (**E**), sarcomere width in μm (**F**), and mitochondrial perimeter in μm (**G**). The number of examined fields of view in the groups varies from 40 to 50.

Figure 4A–D shows representative photomicrographs of the skeletal muscle from four experimental groups. The skeletal muscles of WT mice are characterized by a high-ordered packing of myofibrils. Mitochondrial accumulations are more abundant in the subsarcolemmal regions, and interfibrillar spaces show a more ordered arrangement of organelles. As a rule, paired mitochondria with densely packed cristae are located on both sides of the sarcomere Z disks.

The *mdx* group is distinguished by the heterogeneity of the intracellular organization, which consists of a decrease in the ordering of the myofibril packing, frequent splitting of myofibrillar bundles, irregularities, displacements, and transverse Z-disk deformations. We noted a significant reduction in the width of the sarcomeres compared to WT mice (Figure 4F), which is consistent with the known data [16]. The mitochondrial compartment shows significant changes. The size and shape of both subsarcolemmal and interfibrillar mitochondria are more variable, while the matrix is more osmophilic, and the cristae show irregular packing. Among normal mitochondria, we noted some swollen organelles, whose cristae are distant from each other leading to a decrease in the density of the space between them. In some cases, the outer mitochondrial membrane is damaged. The sarcolemma contains dilated cisterns of the sarcoplasmic reticulum, glycogen, and accumulations of free ribosomes and polyribosomes.

Alisporivir administration has no significant effect on the skeletal muscle structure of WT mice (Figure 4B), also showing a well-ordered packing of myofibrils. At the same time, we noted a significant increase in the size of sarcomeres in this group of animals (Figure 4E,F). At the mitochondrial level, there is a sharp decrease in the size of the organelles (Figure 4G).

The effect of alisporivir is more pronounced in the case of *mdx* animals. The *mdx*+Ali group has a more ordered myofibril packing approaching the control group (Figure 4D). There is a significant increase in the average size of sarcomeres (Figure 4E,F), which is also in line with previous data [16]. The structure of mitochondrial compartments is also similar to the control group; however, the accumulations of mitochondria are less dense in the subsarcolemmal region. In this case, we also noted a significant decrease in the average size of organelles compared to control dystrophin-deficient animals (Figure 4G).

### 2.3. Alisporivir Desensitizes the PTP to Ca^2+^ and Improves the Functioning of Skeletal Muscle Mitochondria in mdx Mice

**Figure 5:**
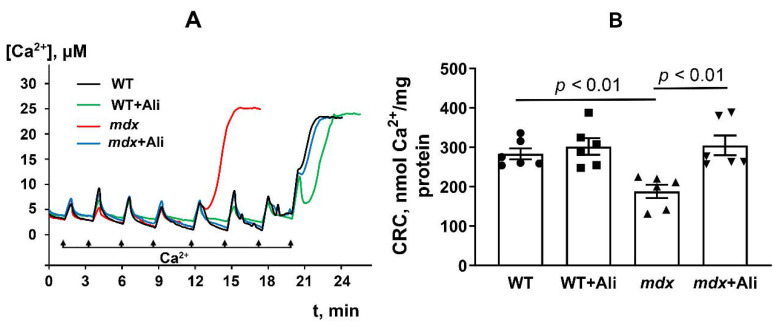
Changes in the external [Ca^2+^] upon successive addition of small Ca^2+^ doses (10 μM) to the suspension of skeletal muscle mitochondria of experimental animals (**A**). Ca^2+^ retention capacity of skeletal muscle mitochondria of experimental animals (**B**). The data are presented as means ± SEM (*n* = 6).

Alisporivir is an inhibitor of the mitochondrial calcium-dependent pore. Therefore, in the next part of the work, we studied the effect of this agent on the resistance of the skeletal muscle mitochondria of mice to the induction of this process. Pore opening was assessed by measuring the Ca^2+^ capacity of mitochondria—the amount of Ca^2^ released upon permeability transition. As we have shown earlier [8,9] and confirmed in this work, *mdx* mice are characterized by a 1.5-fold decrease in the Ca^2+^ capacity of skeletal muscle mitochondria, compared to mitochondria of wild-type animals (Figure 5).

**Figure 6:**
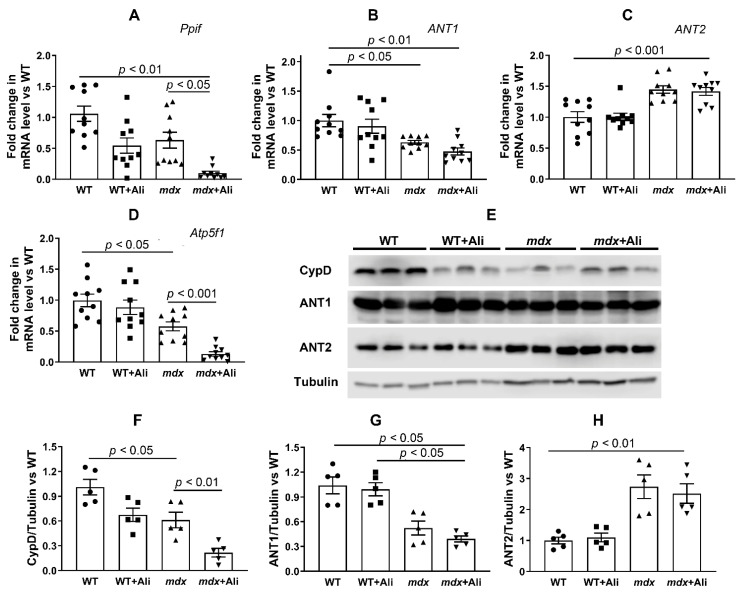
The effect of alisporivir on the content of putative protein components of MPT pore in the skeletal muscle mitochondria. Gene expression of MPT-related proteins measured by real-time PCR: CypD (**A**), ANT1 (**B**), ANT2 (**C**), and α subunit of ATP synthase (**D**). The data are presented as means ± SEM (*n* = 10). Data from the Western blot analysis (**E**). Relative contents of MPT-related proteins, quantification of CypD/tubulin ratio (**F**), ANT1/tubulin ratio (**G**), and ANT2/tubulin ratio (**H**). The data are presented as means ± SEM (*n* = 5).

**Figure 7:**
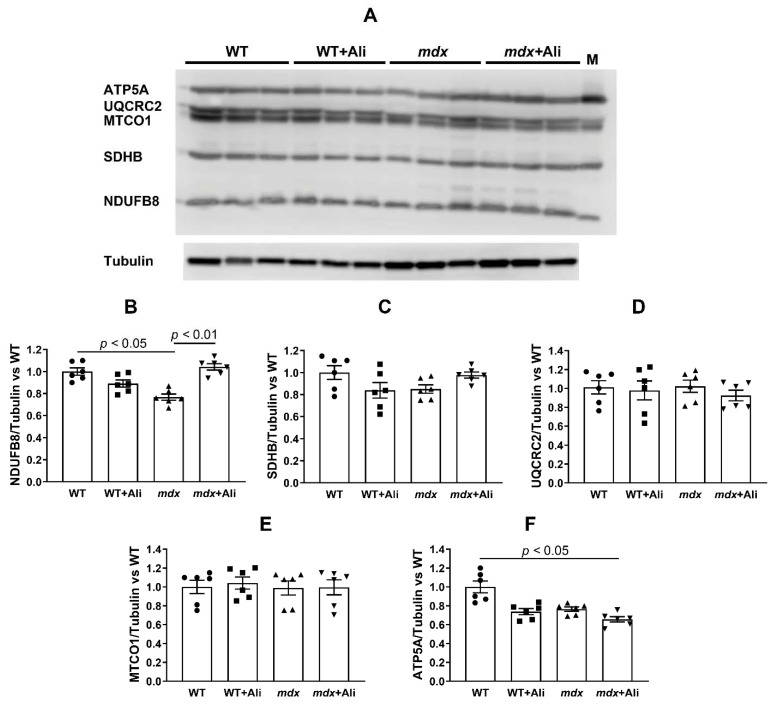
The effect of alisporivir on the content of the proteins of mitochondrial respiratory chain complexes. Data of Western blot analysis (**A**). The letter “M” indicates a positive control (rat heart tissue lysate; mitochondrial extract). Quantification of complex I/tubulin ratio (**B**), complex II/tubulin ratio (**C**), complex III/tubulin ratio (**D**), complex IV/tubulin ratio (**E**), and complex V/tubulin ratio (ATP synthase) (**F**). The data are presented as means ± SEM (*n* = 6).

This indicates a decrease in the resistance of skeletal muscle mitochondria of dystrophin-deficient mice to MPT pore opening. One of the reasons for this may be a change in the content of mitochondrial proteins involved in pore formation. Indeed, we have previously found that the skeletal muscle mitochondria of dystrophin-deficient animals show a significant increase in the level of the ANT2 protein [8,9], which is assumed to be one of the putative protein components of the MPT pore [10]. In this work, we evaluated the effect of alisporivir-based therapy on the content of this protein in the skeletal muscle mitochondria, as well as on the level of other putative protein components of the MPT pore: ANT1, cyclophilin D, and ATP synthase [10]. Figure 6 shows that 12-week dystrophin-deficient mice are characterized by an increase in ANT2 gene expression, compared to wild-type animals. At the same time, we noted a decrease in the expression of genes encoding ANT1, cyclophilin D, and the α-subunit of ATP synthase in *mdx* mice mitochondria, which is in agreement with previously obtained data [8]. In this case, alisporivir treatment leads to a significant decrease in the expression of genes encoding cyclophilin D and the α-subunit of ATP synthase in dystrophin-deficient animals, while the levels of ANT1 and ANT2 do not change. Moreover, we found a significant decrease in the expression of cyclophilin D in WT animals treated with alisporivir. The results obtained are generally confirmed by the Western blot analysis (Figure 6E–H and Figure 7F); however, in this case, the level of the α-subunit of ATP synthase in alisporivir-treated *mdx* mice does not change, and moreover, the reduction of this protein is detected in wild-type mice. One could assume that alisporivir contributes to the normalization of the resistance of skeletal muscle mitochondria in dystrophin-deficient mice to the MPT pore opening, but its effect is not associated with a change in the level of proteins, i.e., putative channel components of the MPT pore. At the same time, the effect of alisporivir may also be due to a decrease in the level of cyclophilin D initiating the assembly of the pore channel, as well as pharmacological blocking of its activity.

We also evaluated the effect of alisporivir on the parameters of respiration and oxidative phosphorylation of the skeletal muscle mitochondria in mice from four experimental groups—a function necessary to maintain muscle energy supply. It is well known that the skeletal muscle mitochondria of *mdx* mice are characterized by a significant decrease in the intensity of respiration and oxidative phosphorylation [4,7,8,9,20]. Indeed, mitochondria of dystrophin-deficient animals show a decrease in the rate of glutamate/malate-fueled respiration in states 3 and 3U_DNP_ (both by 1.4 times), compared to wild-type animals (Table 1). We determined the respiratory control (state 3/state 4) and ADP/O ratios reflecting the efficiency of ATP synthesis by mitochondria. One can see that the skeletal muscle mitochondria of *mdx* mice show a 1.3-fold decrease in respiratory control ratio compared to the WT group. All this suggests that the development of Duchenne dystrophy is associated with a decrease in the efficiency of oxidative phosphorylation in skeletal muscle mitochondria. In this case, alisporivir treatment leads to the normalization of the functional activity of mitochondria. The skeletal muscle mitochondria of alisporivir-treated *mdx* mice show a significant increase in the respiration rate in states 3 and 3U_DNP_ to the values of healthy animals (Table 1). In addition, we noted a significant increase in the respiratory control ratio of mitochondria in dystrophin-deficient animals to that of WT mice. To determine the cause of the change in the rate of respiration of mitochondria, we estimated the content of proteins that build up the complexes of the respiratory chain in the mitochondria of four experimental groups by Western blotting. Twelve-week *mdx* mice show a significant decrease in the content of complex I of the mitochondrial respiratory chain, as well as a reduction in ATP synthase, which is consistent with the known data [4,9] and may underlie the suppression of respiration and oxidative phosphorylation in the mitochondria of dystrophin-deficient animals (Figure 5). Administration of alisporivir normalizes the level of complex I in the skeletal muscle mitochondria of *mdx* mice to the level of wild-type animals, which seems to promote the restoration of respiration intensity and the efficiency of oxidative phosphorylation in these animals. We also noted a decrease in the level of ATP synthase in alisporivir-treated WT animals. At the same time, the levels of other complexes of the respiratory chain of organelles do not change.

**Figure 8:**
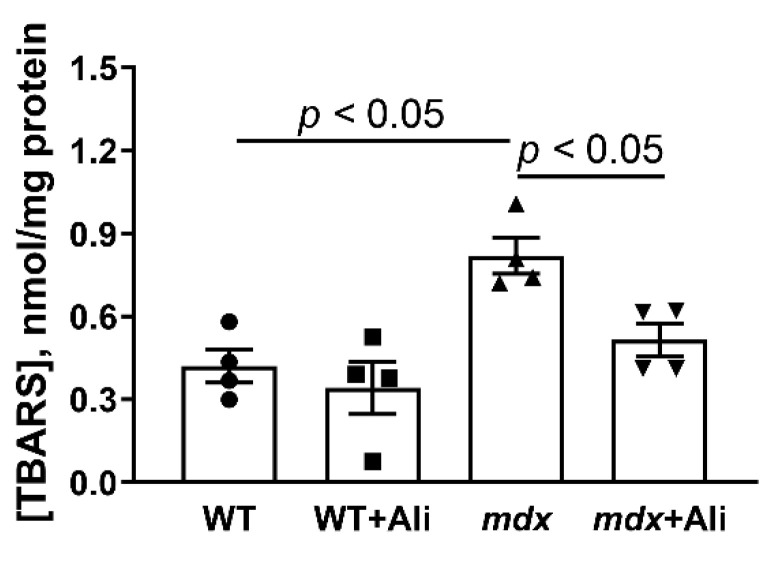
The effect of alisporivir on lipid peroxidation in mitochondria. Lipid peroxidation was assessed by the level of TBARS in the skeletal-muscle mitochondria of experimental groups of animals. The data are presented as means ± SEM (*n* = 4).

The development of DMD is well known to be associated with ROS overproduction in injured skeletal muscles [7,21]. Mitochondria are the main sources of ROS [22], and their excessive production contributes to oxidative damage to organelles and other structures of the cell and muscle fiber in general. In particular, one can see that the skeletal muscle mitochondria of *mdx* mice show a 1.8-fold increase in thiobarbituric reactive substances (malondialdehyde), compared to wild-type animals, indicating an intensification of mitochondrial membrane lipid peroxidation (Figure 8). In this case, alisporivir treatment of *mdx* mice results in a decrease in mitochondrial malondialdehyde level. This may also indicate the ability of alisporivir to reduce the intensity of oxidative stress and oxidative damage to the structures of myocytes in dystrophin-deficient animals.

### 2.4. Alisporivir Suppresses Mitochondrial Dynamics and Biogenesis in Skeletal Muscle

**Figure 9:**
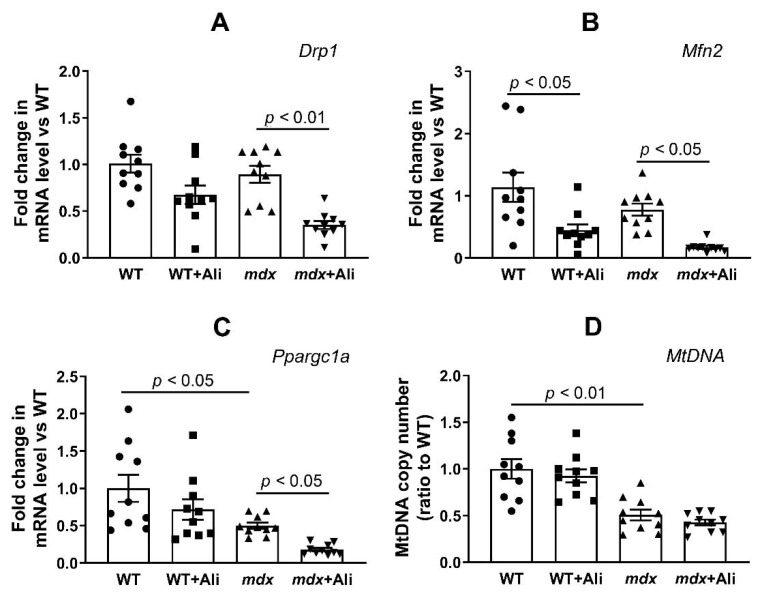
The effect of alisporvir on mitochondrial dynamics and biogenesis in skeletal muscle. The relative mRNA levels of *Drp1* (**A**), *Mfn2* (**B**), *Ppargc1a* (**C**), and mtDNA level (**D**) in the skeletal muscle of experimental animals. The data are presented as means ± SEM (*n* = 10).

It is known that DMD is accompanied by inhibition of mitochondrial biogenesis and changes in the dynamics of organelles (processes of fission and fusion) [23,24,25]. Taking this into account, as well as the important role of cyclophilin D in the regulation of mitochondrial dynamics [26], we evaluated the effect of alisporivir on the expression of proteins responsible for these processes. Here, we studied the expression of genes (*Drp1, Mfn2*, and *Ppargc1a*) encoding proteins responsible for mitochondrial fission, fusion, and mitochondrial biogenesis, respectively. Figure 9 shows that *mdx* mice and wild-type animals show similar levels of *Drp1* and *Mfn2* expression, but dystrophin-deficient mice exhibit a significant reduction in the expression of the transcription factor *Ppargc1a*, indicating suppression of mitochondrial biogenesis. In addition, we noted a decrease in the level of mitochondrial DNA in the skeletal muscles of *mdx* mice, which is also consistent with the known data [25] and indicates a significant decrease in the number of organelles in DMD. In this case, alisporivir treatment is accompanied by a decrease in the expression of both *Drp1* and *Mfn2* indicating the suppression of mitochondrial dynamics. Moreover, we noted a further decrease in *Ppargc1a* expression in alisporivir-treated mice. Additionally, this process is found in both *mdx* mice and wild-type animals, although there is rather a tendency in the latter case. This indicates a further decrease in organelle biogenesis under the action of alisporivir, although it is not associated with a decrease in the level of mtDNA in the skeletal muscles of experimental groups of mice.

## 3. Discussion

Currently, the most common treatment option for DMD is the administration of a subclass of glucocorticoids, in particular, prednisone and its oxazoline derivative deflazacort, which helps to suppress the immune response and inflammatory processes in muscle tissue [27,28]. Such therapy modestly improves muscle strength and cardiopulmonary function [28] but is often associated with significant side effects, as well as impairment of the expression of genes involved in muscle degradation and regeneration [29], leading to chronic myopathies that contribute to proximal muscle weakness [30]. Myostatin inhibition and/or Sarepta’s micro-dystrophin gene therapy show promising results but are only suitable for certain types of mutations [31,32].

The data of numerous studies show that a promising auxiliary strategy for maintaining DMD patients may be an improvement in the functioning of the bioenergetic apparatus of muscle fibers, represented primarily by mitochondria. In particular, suppression of the calcium-dependent MPT pore opening in the inner membrane of organelles is able to prevent the collapse of membrane potential and release of necrosis factors, maintaining the normal functioning of these organelles, including ATP synthesis required for muscle contraction [6,14,15,16]. This can be achieved by knocking out the cyclophilin D gene encoding the main regulatory protein of the mitochondrial matrix capable of starting the process of assembly of the MPT pore channel [14], as well as by pharmacological blocking of its activity using specific inhibitors [6,15,16,17]. Currently, several cyclophilin D inhibitors have been identified. The first such compound is CsA, a fungal cyclic undecapeptide first isolated in 1971 [33], which exhibits a desensitizing effect on MPT pore opening in vitro, as well as in some *in vivo* models [1], but it has no beneficial effect on the state of DMD patients [34], which seems to be related to its immunosuppressive properties, as well as the ability to inhibit calcineurin signaling secondarily reducing myotube differentiation and muscle regeneration. At the beginning of the 21st century, its analog alisporivir (or Debio025), which does not possess immunosuppressive properties, was synthesized. This agent significantly improves pathology in preclinical animal models and biopsies from DMD patients, reducing the level of fibrosis in skeletal muscles and the intensity of necrosis and inflammation [6,15,16]. Indeed, one can see that intraperitoneal administration of alisporivir already at a concentration of 5 mg/kg/day for 4 weeks reduces the intensity of the inflammatory process in dystrophin-deficient *mdx* mice, as evidenced by a decrease in serum creatine kinase, AST, and LDH (Figure 1). Moreover, mice treated with alisporivir show significant improvement in muscle function (Figure 2) compared to control *mdx* mice. It has previously been shown that subcutaneous administration of alisporivir at a dose of 50 mg/kg/day for 6 weeks also has a beneficial effect on the skeletal muscle of *mdx* mice [16]. Thus, one could assume that the effective dose of the drug with this route of administration can be reduced by at least 10 times to achieve a significant effect. It should be noted that we did not find any effect of therapy on pseudohypertrophy in *mdx* mice, which confirms the thesis of the need for early initiation of therapy, prior to rapid weight gain (Figure 3) [16]. At the same time, alisporivir significantly suppressed weight gain and growth in wild-type mice indicating the effect of this agent on the growth of muscle tissue rather than connective tissue fibers replacing it in the case of DMD.

The beneficial effects of alisporivir are also seen at the ultrastructural level of muscle fiber organization. Indeed, one can see that alisporivir-treated *mdx* mice show a normalization of sarcomere size, which is significantly reduced in *mdx* mice compared to WT animals (Figure 4E,F). Moreover, alisporivir also causes an increase in the size of sarcomeres in wild-type mice. We also noted changes in mitochondria, the main targets of alisporivir. In this case, alisporivir treatment show a trend towards normalization of the morphology and structure of *mdx* mice mitochondria, however, the average organelle size decreases markedly (Figure 4G). Moreover, this tendency is also found in the case of alisporivir-treated wild-type mice.

The main effect of alisporivir in mitochondria is the inhibition of the MPT pore opening, leading to an increase in the organelle’s ability to accumulate calcium ions in the matrix. Indeed, one can see that the skeletal muscle mitochondria of alisporivir-treated *mdx* mice show normalization of the calcium capacity parameter, compared to dystrophin-deficient control animals (Figure 5). This effect of alisporivir may be associated with a direct blocking of the activity of cyclophilin D. However, it can also be noted that the action of alisporivir is accompanied by a further significant decrease in the expression of this protein in the skeletal muscles of *mdx* mice (Figure 6A,E,F). In this case, wild-type animals treated with alisporivir also show a decrease in the expression of this regulatory protein (Figure 6A,E,F), but this is not accompanied by an increase in the calcium capacity of skeletal muscle mitochondria (Figure 5). This may indicate the maximum value of this parameter in wild-type animals.

Adenine nucleotide translocator isoforms and ATP synthase are considered as possible channel-forming components of the MPT pore [10]. Their level changes significantly in DMD. In particular, we noted a significant reduction in ANT1 and ATP synthase in the skeletal muscle mitochondria of *mdx* mice, and, on the contrary, a significant increase in the level of ANT2 protein, compared to wild-type mice (Figure 5). The latter protein is practically not expressed in healthy skeletal muscles [35] and its enrichment, as we have shown earlier [8,9], may underlie an increase in the sensitivity of *mdx* mice mitochondria to MPT pore opening. Alisporivir administration has no effect on the level of ANT1 and ANT2 in the mitochondria of both groups of mice, but they show a tendency toward a decrease in the expression of ATP synthase, which is more pronounced in the case of *mdx* mice.

Dysregulation of calcium homeostasis in the skeletal muscle mitochondria of *mdx* mice has a significant effect on the oxidative synthesis of ATP required for muscle contraction. Indeed, the mitochondria of *mdx* mice show significant inhibition of oxidative phosphorylation, as evidenced by a decrease in the respiration rate in the states 3 and 3U_DNP_, as well as in the respiratory control ratio (Table 1). This may be due to both a decrease in the rate of transport of adenine nucleotides, mediated by the reduction of ANT1 (Figure 6), and a reduction of complexes of the respiratory chain of organelles—ATP synthase (Figure 6D and Figure 7F) and complex I (Figure 7B). Alisporivir treatment results in the normalization of mitochondrial respiration (Table 1), which seems to be associated with an increase in the respiratory chain complex I content (Figure 7B). Indeed, in this case, alisporivir does not affect the level of ANT1 (Figure 6B,G) and, moreover, decreases the level of ATP synthase (Figure 6D and Figure 7F), which is also noted in the case of wild-type mice. The latter may cause some tendency toward a decrease in the respiration rate (states 3 and 3U_DNP_) of mitochondria in alisporivir-treated WT animals (Table 1). In the case of *mdx* mice, the normalization of respiration can be associated, first of all, with the enrichment in complex I due to the increase in the amount of Ca^2+^ in the mitochondrial matrix promoting the activation of mitochondrial dehydrogenases and the accumulation of NADH, the substrate of complex I. However, we cannot exclude the effect of alisporivir on the content of lipids (cardiolipin) and electron carriers in the membrane (as coenzyme Q) regulating OXPHOS intensity.

It is important to note that alisporivir also alleviates oxidative stress in the skeletal muscle of *mdx* mice, which is an important destructive factor in DMD, as evidenced by a decrease in the level of lipid peroxidation products (malondialdehyde) in mitochondrial membranes (Figure 8).

All of the above generally indicates a complex positive effect of alisporivir on the functioning of skeletal muscle mitochondria and the state of skeletal muscles in DMD, mediated by inhibition of the activity of cyclophilin D, MPT pore opening, and normalization of calcium homeostasis in organelles. Along with this, we noted the effect of alisporivir on mitochondrial networking indicating some negative aspects of such therapy. Firstly, alisporivir treatment suppresses mitochondrial biogenesis (*Ppargc1a* reduction) in skeletal muscle, which is most pronounced in the case of *mdx* mice (Figure 9C). This is especially important given the already low level of organelle biogenesis and the level of mtDNA in the skeletal muscles of *mdx* mice, compared to WT animals. Secondly, we also noted a decrease in the expression of *Drp1* and mitofusin 2 responsible for mitochondrial fission and fusion in skeletal muscles of both groups of alisporivir-treated mice. Thus, alisporivir also suppresses organelle dynamics, decreasing the episodes of fission and fusion, which is associated with a marked reduction in organelle size (Figure 4G). Recently, it was shown that the target of alisporivir, cyclophilin D, controls the phosphorylation of Drp1 and regulates the recruitment of this protein in the processes of organelle fission and knockout of cyclophilin D or inhibition of its activity by CsA leads to a decrease in the intensity of mitochondrial fission [26]. Moreover, it was shown that CsA reduces the expression of PGC-1a in HepG2 cell culture [36]. Possibly, its non-immunosuppressive analog alisporivir has a similar effect. In particular, we recently found a decrease in cardiac mtDNA levels in mice treated with 2.5 mg/kg alisporivir [37]. These results indicate the complex effect of alisporivir on the functioning of mitochondria, their dynamics, and biogenesis, and again demonstrate the key role of the activity and level of cyclophilin D in the regulation of mitochondrial processes in health and disease.

## 4. Materials and Methods

### 4.1. Animals

C57BL10 mice (wild-type, WT) and dystrophin-deficient C57BL/10ScSn-mdx animals (*mdx* mice) were purchased from Animal Breeding Facility, Branch of the Shemyakin and Ovchinnikov Institute of Bioorganic Chemistry RAS (IBCh RAS Unique Research Device “Bio-model”). After 72 h of acclimatization, *mdx* and wild-type mice were divided into four treatment groups (*n* = 10 per group): (1) vehicle-treated wild-type mice (WT); (2) WT + alisporivir (WT+Ali); (3) *mdx* mice; (4) *mdx* mice treated with alisporivir (*mdx*+Ali). We treated *mdx* and wild-type mice beginning at 8 weeks of age. Alisporivir (1 mg/mL, Medchemexpress, Monmouth Junction, NJ, USA, cat. no. HY-12559) was dissolved in a mixture of DMSO, ethanol, and sterile saline (12.5:25:62.5 *v*/*v*%) and administered in doses of 150–200 μL (5 mg/kg body weight) per mouse interperitoneally every day for up to 4 weeks. Sham-injected controls received solvent alone. At the end of the treatment period, all mice were sacrificed. Blood was collected at the end of all studies for analysis of creatine kinase (CK), lactate dehydrogenase (LDH), and aspartate aminotransferase (AST) levels using the appropriate reagent kits (Vector-Best, Novosibirsk, Russia). Mitochondrial isolation was performed in fresh samples of skeletal muscle tissue (quadriceps of both hindlimbs). The rest of the tissue was stored at −80 °C until analyzed.

### 4.2. Wire-Hanging Test

Muscle function and endurance of the mice were assessed using a wire-hanging test. In this case, each mouse was placed on a 3 mm string (38 cm long and 49 cm above a soft surface to cushion animals that fall off). The mouse was held on the string by its front paws and hung for 30 s. Scoring the test results was carried out according to a well-known approach [38]: hanging for 1–5 s = 1, hanging for 6–10 s = 2, hanging for 11–20 s = 3, hanging for 21–30 s = 4, hanging for 30 s = 5. Placing one forepaw on a bar support without falling = 5. An animal that repeatedly failed before the 5 s elapsed received only 1 point. Each mouse was allowed three attempts, and the average was used for the final calculation.

### 4.3. Electron Microscopy

For the electron microscopy examination, pieces of the skeletal muscle (quadriceps, two samples in each experimental group) were taken and fixed for 2 h in 0.1 M phosphate-buffered saline (PBS, pH 7.4) supplemented with 2.5% glutaraldehyde. Further, the tissue was fixed in PBS containing 1% osmic acid, and water was removed by increasing the alcohol concentration. The resulting skeletal muscle samples were encapsulated in Epon 812 resin. A Leica EM UC6 microtome (Leica Microsystems, Wetzlar, Germany) was used to prepare ultrathin sections (70–75 nm) and then stained using lead citrate and uranyl acetate. The samples were evaluated and imaged using a JEM-100B electron microscope (JEOL, Tokyo, Japan). Negative images digitized with an Epson V700 scanner were used for morphometric analysis with the Image Tool 3.0 software, which included an assessment of the size of sarcomeres and mitochondria.

### 4.4. Mitochondria Isolation and Determination of Respiration and Oxidative Phosphorylation

Mitochondria were isolated from skeletal muscle tissue (quadriceps of both hindlimbs) by differential centrifugation, as described earlier [8,9]. The isolation medium contained 67 mM Sucrose, 50 mM KCl, 10 mM EDTA, 0.2% BSA, and 50 mM Tris/HCl buffer (pH 7.4). The resulting suspension of mitochondria was resuspended in 250 mM Sucrose and 10 mM Tris/HCl buffer (pH 7.4) and contained 20–30 mg of mitochondrial protein/mL, as determined by the Lowry method. The rate of oxygen consumption was measured polarographically with a Clark-type gold electrode and Oxygraph-2k (Oroboros Instruments, Innsbruck, Austria) at 25 °C under continuous stirring. The reaction medium contained 120 mM KCl, 5 mM NaH_2_PO_4_, 2.5 mM potassium malate, 2.5 mM potassium glutamate, 10 mM Hepes/KOH, pH 7.4. Other reagents: 0.2 mM ADP and 50 μM 2,4-dinitrophenol (DNP). The assessment of the functional parameters of mitochondrial respiration was carried out by the generally accepted method [39]. The concentration of mitochondrial protein was 0.25 mg/mL.

### 4.5. Determination of Ca^2+^ Retention by Mitochondria, MPT Pore Opening Assay

Mitochondrial Ca^2+^ transport was recorded using the Arsenazo III dye at 675–685 nm and a Tecan Spark 10M plate reader (Tecan Group Ltd, Männedorf, Switzerland) as described previously [8,9]. To determine the ability of mitochondria (0.25 mg of mitochondrial protein/mL) to retain Ca^2+^, 10 μM CaCl_2_ was successively added into the reaction medium 210 mM mannitol, 70 mM sucrose, 1 mM KH_2_PO_4_, 2.5 mM potassium malate, 2.5 mM potassium glutamate, 50 μM arsenazo III, 10 μM EGTA, and 10 mM HEPES-KOH (pH 7.4). The massive release of calcium from organelles after several external calcium supplements was evaluated as MPT pore opening.

### 4.6. Lipid Peroxidation

The intensity of lipid peroxidation in skeletal muscle mitochondria was assessed by thiobarbituric acid-reactive substances (TBARS) assay, sensitive to malondialdehyde and other minor aldehyde forms [40].

### 4.7. RNA Extraction, Reverse Transcription, and Quantitative Real-Time PCR

Total RNA was isolated from 100 mg of deep-frozen tissue samples using an ExtractRNA kit (#BC032, Eurogen, Moscow, Russia). DTLite5 amplifier (DNA-Technologies LLC, Moscow, Russia) and qPCRmix-HS SYBR reaction mixture (Evrogen, Moscow, Russia) were used for real-time PCR. Gene-specific primers were selected and analyzed using Primer-BLAST [41] (Table 2 shows the sequences used). The mRNA level of *Rplp2* was used to normalize the expression of each gene. The quantitative assessment of the results was carried out using the comparative C_T_ method [42].

### 4.8. Analysis of mtDNA/nDNA Ratio

A total of 10 mg of skeletal muscle tissue was used to extract total DNA (nuclear and mtDNA) using a DNA-Extran 2 kit (Sintol, Moscow, Russia). The reaction used 1 ng of total DNA. The content of mtDNA in skeletal muscles was estimated by PCR as described previously [43] and expressed as mtDNA/nuclear DNA ratio. The ND4 gene of the mouse mitochondrial genome and the nuclear GAPDH gene were used for analysis. Their ratio reflected the number of mtDNA copies to the number of nDNA copies. Table 2 shows the primers for mtDNA and nDNA estimation. DTLite5 amplifier (DNA-Technologies LLC, Moscow, Russia) and qPCRmix-HS SYBR reaction mixture supplemented with fluorescent DNA binding dye SYBR Green II (Evrogen, Moscow, Russia) were used for real-time PCR.

### 4.9. Electrophoresis and Immunoblotting of Mitochondrial OXPHOS and MPT Pore Proteins

Total protein extracts were prepared from 10 mg of the frozen muscle. To maintain extract integrity and function, complete protease inhibitor cocktail (P8340, Sigma-Aldrich, USA), phosphatase inhibitor cocktail 3 (P0044 Sigma-Aldrich, St. Louis, MO, USA), PMSF (1 mM), Na_3_VO_4_ (1 mM), EGTA (1 mM), and EDTA (1 mM) were used. Proteins were isolated using a RIPA buffer (20–188, Merck Millipore Ltd., Billerica, MA, USA). Quick Start Bradford Protein Assay (Bio-Rad Laboratories, Hercules, CA, USA) was used to quantify protein content. The samples were diluted in Laemmli buffer, run on 12.5% SDS–PAGE (10 µg/lane), and transferred to a 0.45 µm nitrocellulose membrane (Cytiva, Marlborough, MA, USA). After overnight blocking, the membrane was incubated with the appropriate primary antibody. The total OXPHOS Rodent WB antibody cocktail (ab110413), anti-cyclophilin F (CypD) (ab64935), anti-ANT1 (ab102032) and anti-alpha tubulin antibody (ab4074) were from Abcam. The monoclonal rabbit anti-ANT2/SLC25A5 (#14671) antibody was purchased from Cell Signalling Technology, Inc. The corresponding secondary horseradish peroxidase-conjugated antibodies (7074, Cell Signaling technology Inc., (Danvers, MA, USA) were used to detect immunoreactivity. Chemiluminescent ECL reagents (Pierce, Rockford, IL, USA) were used to determine the peroxidase activity. Proteins were visualized and quantified using the LI-COR system (LI-COR, Lincoln, NE, USA) and normalized to the alpha tubulin used as a loading control. Optical density measurements were performed by LI-COR Image Studio software.

### 4.10. Statistical Processing of Data

Results are shown as mean ± standard error of the mean (m ± SEM). GraphPad Prism version 6.0 for Windows (GraphPad Software Inc., La Jolla, CA, USA) was used for statistical data analysis. One-way repeated analysis of variance (ANOVA), followed by Tukey’s post hoc test, was used to assess the statistical significance of differences between treatment groups. Differences were indicated as statistically significant in the case of *p* < 0.05.

## 5. Conclusions

The data obtained in this work suggest that a decrease in the activity and expression of cyclophilin D by alisporivir (MPT pore inhibitor) treatment restores the structure and functions of skeletal muscle mitochondria in dystrophin-deficient *mdx* mice, improves the functioning of skeletal muscles, and reduces the intensity of destructive processes. We demonstrated that administration of alisporivir at a concentration of 5 mg/kg/day (1) restores the ultrastructure of the skeletal muscle mitochondria in *mdx* animals and (2) restores the calcium retention capacity and respiratory control ratio, and also reduces the lipid peroxidation intensity. In the short term, this option may have a beneficial effect on the state of DMD animals, which has also been shown in cyclophilin D null (*Ppif*^−/−^) models. At the same time, modulation of cyclophilin D by alisporivir is accompanied by a decrease in the activity of mitochondrial biogenesis and the dynamics of organelles. One could speculate that in the long term, suppression of cyclophilin D may adversely affect the growth and differentiation of muscle fibers and other tissues, as seen in the case of alisporivir-treated wild-type animals. In this regard, it is possible that the combination of alisporivir and the histone deacetylase inhibitor givinostat reversing the suppression of mitochondrial biogenesis in DMD [25] may contribute to addressing this issue. Nevertheless, we can summarize that the MPT pore targeting approach may be used as an effective adjunctive strategy in the treatment of DMD.

## Figures and Tables

**Table 1:** The effect of alisporivir treatment on the parameters of respiration and oxidative phosphorylation of skeletal muscle mitochondria of studied groups of mice.

Animal (*n* = 10)	State 2	State 3	State 4	State 3U_DNP_	RC	ADP/O
	nmol O_2_/min per 1 mg of Protein				Relative Units	
WT	19.6 ± 1.0	172.7 ± 6.7	26.2 ± 1.1	213.4 ± 10.7	6.6 ± 0.1	3.1 ± 0.3
WT+Ali	17.9 ± 0.8	156.4 ± 7.1	23.0 ± 0.6	202.0 ± 12.5	6.8 ± 0.2	2.9 ± 0.1
*mdx*	17.1 ± 1.3	127.9 ± 10.0 *	25.1 ± 1.3	150.4 ± 13.2 *	5.1 ± 0.2 *	3.2 ± 0.2
*mdx+*Ali	21.0 ± 0.8	164.4 ± 3.3	28.7 ± 1.3	201.9 ± 7.6	5.8 ± 0.2 *#	2.9 ± 0.1

Medium composition: 120 mM KCl, 5 mM NaH_2_PO_4_, and 10 mM HEPES-KOH buffer (pH 7.4). Respiration of mitochondria was fueled by 2.5 mM glutamate + 2.5 mM malate. Respiration of mitochondria in state 3 was initiated by 200 μM ADP. The data are presented as means ± SEM. * *p* < 0.05 versus other groups. # *p* < 0.05 versus *mdx* group.

**Table 2:** List of gene-specific primers for RT-PCR analysis.

Gene	Forward (5‘ → 3’)	Reverse (5’ → 3’)
* Ant1 *	CTATGACACTGCCAAGGGGATG	TCAAACGGATAGGACACCAGC
* Ant2 *	TCTGGACGCAAAGGAACTGA	GACCATGCGCCCTTGAAA
* Ppif *	GCAGATGTCGTGCCAAAGACTG	GCCATTGTGGTTGGTGAAGTCG
* Drp1 *	TTACAGCACACAGGAATTGT	TTGTCACGGGCAACCTTTTA
* Mfn2 *	CACGCTGATGCAGACGGAGAA	ATCCCAGCGGTTGTTCAGG
* Ppargc1a *	CTGCCATTGTTAAGACCGAG	GTGTGAGGAGGGTCATCGTT
* Rplp2 *	CGGCTCAACAAGGTCATCAGTGA	AGCAGAAACAGCCACAGCCCCAC
*Nd4*	ATTATTATTACCCGATGAGGGAACC	ATTAAGATGAGGGCAATTAGCAGT
*Gapdh*	GTGAGGGAGATGCYCAGTGT	CTGGCATTGCTCTCAATGAC

## Data Availability

The data presented in this study are available on request from the corresponding author.

## References

[1] Ignatieva E., Smolina N., Kostareva A., Dmitrieva R. (2021). Skeletal muscle mitochondria dysfunction in genetic neuromuscular disorders with cardiac phenotype. Int. J. Mol. Sci..

[2] Bodensteiner J.B., Engel A.G. (1978). Intracellular calcium accumulation in Duchenne dystrophy and other myopathies: A study of 567,000 muscle fibers in 114 biopsies. Neurology.

[3] Williams G.S.B., Boyman L., Chikando A.C., Khairallah R.J., Lederer W.J. (2013). Mitochondrial calcium uptake. Proc. Natl. Acad. Sci. USA.

[4] Rybalka E., Timpani C., Cooke M.B., Williams A., Hayes A. (2014). Defects in mitochondrial ATP synthesis in dystrophin-deficient mdx skeletal muscles may be caused by complex I insufficiency. PLoS ONE.

[5] Vila M.C., Rayavarapu S., Hogarth M., van der Meulen J.H., Horn A., Defour A., Takeda S., Brown K.J., Hathout Y., Nagaraju K. (2017). Mitochondria mediate cell membrane repair and contribute to Duchenne muscular dystrophy. Cell Death Differ..

[6] Schiavone M., Zulian A., Menazza S., Petronilli V., Argenton F., Merlini L., Sabatelli P., Bernardi P. (2017). Alisporivir rescues defective mitochondrial respiration in Duchenne muscular dystrophy. Pharmacol. Res..

[7] Hughes M.C., Ramos S.V., Turnbull P.C., Rebalka I.A., Cao A., Monaco C.M., Varah N.E., Edgett B.A., Huber J.S., Tadi P. (2019). Early myopathy in Duchenne muscular dystrophy is associated with elevated mitochondrial H_2_O_2_ emission during impaired oxidative phosphorylation. J. Cachexia Sarcopenia Muscle.

[8] Dubinin M.V., Talanov E.Y., Tenkov K.S., Starinets V.S., Mikheeva I.B., Sharapov M.G., Belosludtsev K.N. (2020). Duchenne muscular dystrophy is associated with the inhibition of calcium uniport in mitochondria and an increased sensitivity of the organelles to the calcium-induced permeability transition. Biochim. Biophys. Acta BBA Mol. Basis Dis..

[9] Dubinin M.V., Talanov E.Y., Tenkov K.S., Starinets V.S., Belosludtseva N.V., Belosludtsev K.N. (2020). The effect of deflazacort treatment on the functioning of skeletal muscle mitochondria in Duchenne muscular dystrophy. Int. J. Mol. Sci..

[10] Belosludtsev K.N., Dubinin M., Belosludtseva N., Mironova G.D. (2019). Mitochondrial Ca^2+^ transport: Mechanisms, molecular structures, and role in cells. Biochem. Mosc..

[11] Bonora M., Patergnani S., Ramaccini D., Morciano G., Pedriali G., Kahsay A.E., Bouhamida E., Giorgi C., Wieckowski M.R., Pinton P. (2020). Physiopathology of the permeability transition pore: Molecular mechanisms in human pathology. Biomolecules.

[12] Porter J.G.A., Beutner G. (2018). Cyclophilin D, somehow a master regulator of mitochondrial function. Biomolecules.

[13] Amanakis G., Murphy E. (2020). Cyclophilin D: An integrator of mitochondrial function. Front. Physiol..

[14] Millay D.P., Sargent M.A., Osinska H., Baines C., Barton E.R., Vuagniaux G., Sweeney H.L., Robbins J., Molkentin J.D. (2008). Genetic and pharmacologic inhibition of mitochondrial-dependent necrosis attenuates muscular dystrophy. Nat. Med..

[15] Reutenauer J., Dorchies O.M., Patthey-Vuadens O., Vuagniaux G., Ruegg U.T. (2008). Investigation of Debio 025, a cyclophilin inhibitor, in the dystrophic mdx mouse, a model for Duchenne muscular dystrophy. Br. J. Pharmacol..

[16] Wissing E.R., Millay D.P., Vuagniaux G., Molkentin J.D. (2010). Debio-025 is more effective than prednisone in reducing muscular pathology in mdx mice. Neuromuscul. Disord..

[17] Stocco A., Smolina N., Sabatelli P., Šileikytė J., Artusi E., Mouly V., Cohen M., Forte M., Schiavone M., Bernardi P. (2021). Treatment with a triazole inhibitor of the mitochondrial permeability transition pore fully corrects the pathology of sapje zebrafish lacking dystrophin. Pharmacol. Res..

[18] Stupka N., Gregorevic P., Plant D.R., Lynch G.S. (2004). The calcineurin signal transduction pathway is essential for successful muscle regeneration in mdx dystrophic mice. Acta Neuropathol..

[19] Hansson M.J., Mattiasson G., Mansson R., Karlsson J.O., Keep M.F., Waldmeier P., Ruegg U.T., Dumont J.M., Besseghir K., Elmér E. (2004). The nonimmunosuppressive cyclosporin analogs NIM811 and UNIL025 display nanomolar potencies on permeability transition in brain-derived mitochondria. J. Bioenerg. Biomembr..

[20] Kuznetsov A.V., Winkler K., Wiedemann F., von Bossanyi P., Dietzmann K., Kunz W.S. (1998). Impaired mitochondrial oxidative phosphorylation in skeletal muscle of the dystrophin-deficient mdx mouse. Mol. Cell. Biochem..

[21] Dubinin M.V., Talanov E.Y., Tenkov K.S., Starinets V.S., Mikheeva I.B., Belosludtsev K.N. (2020). Transport of Ca^2+^ and Ca2^+^-dependent permeability transition in heart mitochondria in the early stages of Duchenne muscular dystrophy. Biochim. Biophys. Acta BBA Bioenerg..

[22] Zorov D.B., Juhaszova M., Sollott S.J. (2014). Mitochondrial reactive oxygen species (ROS) and ROS-induced ROS release. Physiol. Rev..

[23] Pant M., Sopariwala D.H., Bal N.C., Lowe J., Delfín D.A., Rafael-Fortney J., Periasamy M. (2015). Metabolic dysfunction and altered mitochondrial dynamics in the utrophin-dystrophin deficient mouse model of Duchenne muscular dystrophy. PLoS ONE.

[24] De Mario A., Gherardi G., Rizzuto R., Mammucari C. (2021). Skeletal muscle mitochondria in health and disease. Cell Calcium.

[25] Giovarelli M., Zecchini S., Catarinella G., Moscheni C., Sartori P., Barbieri C., Roux-Biejat P., Napoli A., Vantaggiato C., Cervia D. (2021). Givinostat as metabolic enhancer reverting mitochondrial biogenesis deficit in Duchenne muscular dystrophy. Pharmacol. Res..

[26] Xiao A., Gan X., Chen R., Ren Y., Yu H., You C. (2017). The cyclophilin D/Drp1 axis regulates mitochondrial fission contributing to oxidative stress-induced mitochondrial dysfunctions in SH-SY5Y cells. Biochem. Biophys. Res. Commun..

[27] Markham A., Bryson H.M. (1995). Deflazacort. Drugs.

[28] Bylo M., Farewell R., Coppenrath V.A., Yogaratnam D. (2020). A review of deflazacort for patients with Duchenne muscular dystrophy. Ann. Pharmacother..

[29] Hanaoka B.Y., Peterson C.A., Horbinski C., Crofford L.J. (2012). Implications of glucocorticoid therapy in idiopathic inflammatory myopathies. Nat. Rev. Rheumatol..

[30] Schakman O., Gilson H., Kalista S., Thissen J. (2009). Mechanisms of muscle atrophy induced by glucocorticoids. Horm. Res..

[31] Morine K.J., Bish L.T., Pendrak K., Sleeper M.M., Barton E.R., Sweeney H.L. (2010). Systemic myostatin inhibition via liver-targeted gene transfer in normal and dystrophic mice. PLoS ONE.

[32] Mendell J.R., Sahenk Z., Lehman K., Nease C., Lowes L.P., Miller N.F., Iammarino M.A., Alfano L.N., Nicholl A., Al-Zaidy S. (2020). Assessment of systemic delivery of rAAVrh74.MHCK7.micro-dystrophin in children with Duchenne muscular dystrophy. JAMA Neurol..

[33] Gams W. (1971). Tolypocladiumeine Hyphomycetengattung mit geschwollenen Phialiden. Persoonia.

[34] Kirschner J., Schessl J., Schara U., Reitter B., Stettner G.M., Hobbiebrunken E., Wilichowski E., Bernert G., Weiss S., Stehling F. (2010). Treatment of Duchenne muscular dystrophy with ciclosporin A: A randomised, double-blind, placebo-controlled multicentre trial. Lancet Neurol..

[35] Stepien G., Torroni A., Chung A., Hodge J., Wallace D. (1992). Differential expression of adenine nucleotide translocator isoforms in mammalian tissues and during muscle cell differentiation. J. Biol. Chem..

[36] Qi R., Wang D., Xing L., Wu Z. (2018). Cyclosporin A inhibits mitochondrial biogenesis in Hep G2 cells. Biochem. Biophys. Res. Commun..

[37] Belosludtseva N.V., Starinets V.S., Mikheeva I.B., Serov D.A., Astashev M.E., Belosludtsev M.N., Dubinin M.V., Belosludtsev K.N. (2021). Effect of the MPT pore inhibitor alisporivir on the development of mitochondrial dysfunction in the heart tissue of diabetic mice. Biology.

[38] Deacon R.M. (2013). Measuring motor coordination in mice. J. Vis. Exp..

[39] Belosludtsev K.N., Belosludtseva N.V., Kosareva E.A., Talanov E.Y., Gudkov S.V., Dubinin M.V. (2020). Itaconic acid impairs the mitochondrial function by the inhibition of complexes II and IV and induction of the permeability transition pore opening in rat liver mitochondria. Biochimie.

[40] Belosludtseva N.V., Starinets V.S., Pavlik L.L., Mikheeva I.B., Dubinin M.V., Belosludtsev K.N. (2020). The effect of S-15176 difumarate salt on ultrastructure and functions of liver mitochondria of c57bl/6 mice with streptozotocin/high-fat diet-induced type 2 diabetes. Biology.

[41] Ye J., Coulouris G., Zaretskaya I., Cutcutache I., Rozen S., Madden T.L. (2012). Primer-BLAST: A tool to design target-specific primers for polymerase chain reaction. BMC Bioinform..

[42] Schmittgen T.D., Livak K.J. (2008). Analyzing real-time PCR data by the comparative C_T_ method. Nat. Protoc..

[43] Quiros P.M., Goyal A., Jha P., Auwerx J. (2017). Analysis of mtDNA/nDNA ratio in mice. Curr. Protoc. Mouse Biol..

